# Polylactic Acid–Glass Fiber Composites: Structural, Thermal, and Electrical Properties

**DOI:** 10.3390/polym14194012

**Published:** 2022-09-25

**Authors:** Teodoro Klaser, Luka Balen, Željko Skoko, Luka Pavić, Ana Šantić

**Affiliations:** 1 Ruđer Bošković Institute, Bijenička cesta 54, 10000 Zagreb, Croatia; 2 Department of Physics, Faculty of Science, University of Zagreb, Bijenička cesta 32, 10000 Zagreb, Croatia

**Keywords:** polylactic acid, glass fibers, PLA composites, thermal properties, electrical properties, reinforcing PLA

## Abstract

The aim of this study is to investigate the influence of different glass fibers made of commercial silicate, borosilicate, and laboratory-made iron–phosphate compositions, on the preparation of polylactic acid (PLA) composites and their structural and physical properties. The thermal, structural, and electrical properties of prepared PLA–glass fiber composites were studied using differential scanning calorimetry, X-ray diffraction, microscopy, and impedance spectroscopy. The structural as well as morphological, thermal, and electrical properties of all PLA–glass composites were found to be very similar and independent of the composition and aspect ratio of glass fibers. All types of glass fibers improve mechanical properties, increase thermal stability, and decrease the electrical conductivity of PLA, thereby producing mechanical strong electrically insulating composite material with potential in various applications.

## 1. Introduction

Over the past decade, significant research efforts have been made towards the development of biopolymers derived from renewable resources to replace environmentally harmful synthetic polymers. One of the most studied biopolymers is polylactic acid (PLA), a versatile biodegradable material derived exclusively from natural resources [1,2]. The combination of properties such as biocompatibility, good biodegradability, high stiffness, and UV stability makes PLA and its composites desirable materials for biomedical applications as well as for the packaging, textile, and automotive industries [2,3,4,5,6]. 

Although PLA offers many advantages in terms of production and performance, its poor mechanical properties, i.e., brittleness and low heat resistance, still pose certain limitations from an application perspective. To overcome these drawbacks and produce an environmentally friendly polymer with superior physiochemical properties, various PLA composites with different fillers have been developed in the last decade. In general, the fillers used for the preparation of polymer composites fall into two categories: natural and synthetic fibers [7]. Natural fibers such as flax, ramie, hemp, and cellulose, although they are fully biodegradable and have no negative environmental impacts, also have disadvantages that are mainly related to high moisture absorption and poor mechanical performance of the composite [5]. On the other hand, synthetic fillers such as glass or carbon fibers are generally much stronger and therefore improve the mechanical properties of polymer [7,8]. In particular, glass fibers are especially attractive due to their good mechanical properties, good thermal resistance, and relatively simple and low-cost production [8,9,10,11]. However, although the application of glass fibers has been extensively studied for the reinforcement of various polymers, their use for reinforcing PLA has been investigated only scarcely [12]. Jaszkiewicz et al. [13] reported a comparative study of the mechanical properties of composites based on PLA and polypropylene, both reinforced with 30% of glass fibers, natural fiber abaca, and manmade cellulose. They showed that all composites based on PLA have improved stiffness in comparison with those based on polypropylene. Further, Varsavas and Kaynak [14] reported that glass fiber reinforcement led to significant increases in the strength and elastic modulus of PLA, and the optimum glass fiber content was 15 wt%. More recently, Wang et al. [7] showed that silane-modified glass fibers improve the mechanical properties, thermal stability, and foaming ability of PLA, thus forming strong PLA composites and their foams with a promising future in preparing lightweight structural components for the automotive and aircraft industries. In their other study [15], they showed that glass fibers can significantly enhance the crystallization of PLA upon heating, thus leading to the improved mechanical properties and thermal stability of PLA–glass fiber composites. Additionally, studies [16,17] in the field of biomaterials show that phosphate-based and bioactive glass fibers, when blended with PLA, form fully degradable composites that can be used for bone repair. 

Generally, the mechanical properties of glass fiber-reinforced PLA depend on the composition, content, aspect ratio, and distribution of fibers, as well as the interaction between fiber and PLA matrix [15]. Since many glass fiber parameters can be varied in the preparation of PLA–glass composites, thorough knowledge of the composition and structural properties is needed to harness their potential. Furthermore, it is important to note that the manufacturing, processing, and industrial applications of glass fibers inevitably generate certain amounts of flawed or waste fiber material. The usage of this waste material for the preparation of composites could be a promising way to recycle them. Therefore, the aim of this study was to investigate the influence of three commercially available types as well as one non-commercial, laboratory-made batch of glass fibers on the structural, thermal, and electrical properties of PLA–glass composites. The results of the characterization of these novel PLA–glass composites using differential scanning calorimetry (DSC), X-ray diffraction (XRD), microscopy, and impedance spectroscopy (IS) show that all investigated glass fibers form compact, mechanically reinforced PLA–glass composites with improved thermal stability and lower electrical conductivity which can be used in many applications.

## 2. Materials and Methods

Industrial-grade PLA, BioBatch1851, was supplied by TechnoCompound GmbH, Bad Sobernheim, Germany. The PLA has a density of 1.39 g/cm^3^ and a melt flow index of 3.5 g/10 min. For preparation of composites we used four types of glass fibers (GF): Hybon^TM^ 2002 roving (HYBG), Alkali Resistant Glass Fiber (ARG), Woven Roving Glass (WG) and iron phosphate glass fibers (IPG) to prepare glass composites. Commercial GF, Hybon™ 2002 roving and Alkali Resistant Glass Fiber roving, were purchased from Nippon Electric Glass Co., Ltd., Tokyo, Japan while Woven Roving Glass was supplied by Kelteks d.o.o., Karlovac, Croatia. The glass fibers of composition 40Fe_2_O_3_-60P_2_O_5_ in mol% (IPG) were prepared in laboratory using the procedure given in reference [18]. The composition of used glass fibers is given in Table 1. The PLA–glass composites were synthesized by dissolving the PLA in a solution with organic solvent and glass fibers. Pellets of polylactic acid (m_(PLA)_ = 1.0 g) were submerged in dichloromethane (V_(DCM)_ ≈ 3 mL) inside a small glass bottle with a cap. The process was carried out in a fume hood. After 24 h, the solution was stirred and mixed with the glass fibers (m_(GF)_ = 1.0 g). Prior to the mixing, the glass fibers were crushed with a mortar and pestle after being heated at 400 °C for 1 h in order to break them into micro size more easily. After mixing, the solution was homogenized for 3 min using an ultrasonic sonicator. The homogenized solution was poured into a small glass beaker (5 cm^3^). It was then left inside the fume hood for another 24 h to allow the dichloromethane to evaporate completely. After that, the samples were ready for characterization.

X-ray diffraction data were collected at RT with Bruker D8 Discover diffractometer (Bruker AXS GmbH, Karlsruhe, Germany) equipped with LYNXEYE XE-T detector. The samples were analyzed in a parallel beam configuration for flat solid samples. The identification of the composition of PLA and composites was made by Eva software and search and match procedure through PDF4 database. 

For thermal analysis, a Netzsch STA 449 F5 Jupiter was used in the temperature range from 20 to 200 °C with a heating rate of 10 K/min. Synthetic air purge with a flow of N_2_/O_2_ of 50/20 mL/min was applied for the measurements and Al_2_O_3_ crucibles were used. The samples were heated once over the mentioned temperature range, cooled near RT temperature after which a second heating cycle was performed. The DSC specimens have approximately a mass of 20 mg. 

PLA–glass composites surface and cross-section morphology was investigated by Nikon Aclipse LV150NL, a polarizing microscope with a digital camera (Optoteam OPTOCAM II), Field Emission Scanning Electron Microscopy (FE-SEM) JEOL, model JSM 7000F (UK) and Thermo Fisher Scientific model Axia™ ChemiSEM™ (Waltham, MA, USA). Samples were not coated with an electrically conductive layer and the accelerating voltage was kept low.

The electrical properties of the prepared composites were investigated with impedance spectroscopy (IS). The real and imaginary parts of complex impedance were measured using an impedance analyzer (Novocontrol Alpha-AN Dielectric Spectrometer, Novocontrol Technologies GmbH & Co. KG, Hundsangen, Germany) over a wide range of frequencies (0.01 Hz to 1 MHz) and temperature from 20 to 200 °C, with the step of 20 °C. For electrical contact, samples with ~1 mm thickness were pressed between brass electrodes, 5 mm in diameter. 

In order to probe whether different types of glasses can enhance the crystallization process of PLA, the PLA–glass composites were annealed at 90 °C for 1 h after being heated at 210 °C for 10 min. After the heat treatment, the annealed samples were analyzed by XRD.

## 3. Results and Discussion

### 3.1. Structure and Morphological Properties of PLA–Glass Fiber Composites

**Figure 1:**
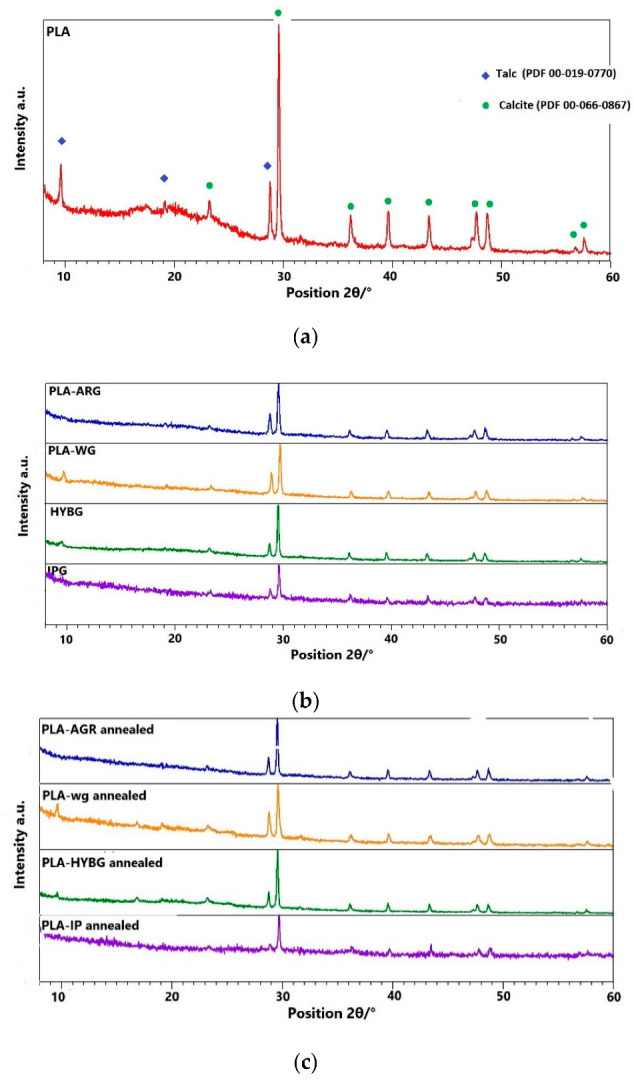
Diffraction patterns of (**a**) neat PLA, (**b**) as prepared and (**c**) heat-treated PLA–glass composites.

The first step in the characterization of the prepared PLA–glass composites was to structurally characterize the starting PLA material. Figure 1a shows the XRD diffractogram of the industrial grade PLA with strong diffraction lines corresponding to impurities of calcite and talc, superimposed on a broad halo attributed to amorphous PLA. Two impurity compounds, calcite and talc, are used as fillers for industrial PLA. The diffractograms of the prepared PLA–glass composites resemble those of PLA, indicating that neither PLA nor glass fibers are crystallized; see Figure 1b. 

All PLA–glass composites comprise a fully amorphous PLA matrix (containing calcite and talc) with embedded glass fibers. To investigate whether the addition of various glass fibers can promote the crystallization of PLA, the composites were heated and held at 210 °C for 10 min and then annealed at 90 °C for 1 h. The XRD patterns of the heat-treated PLA–glass composites show no new diffraction lines, suggesting that the crystallization of PLA was not triggered with any type of glass fibers and, hence all composites remained amorphous, see Figure 1c. This finding is not in agreement with the results reported by Wang et al. [15], who showed that glass fibers significantly accelerate and enhance the crystallization of PLA during heat treatment at 80 °C and 100 °C. However, in the reported study, the surface of glass fibers was modified with 3-aminopropyl methyl dimethoxy silane in order to improve the interaction between glass fibers and PLA. In our study, the glass fiber surface was not modified, which could lead to somewhat weaker interaction between fiber and PLA matrix. Although our study suggests that this difference is not crucial for the macroscopic compactness and mechanical properties of composites, which apparently exhibit significantly higher mechanical strength and stiffness in comparison to the neat PLA, the loose bonding between glass fibers and PLA matrix could be the reason why crystallization was not achieved with thermal treatment in our case. Therefore, it could be inferred that fibers can promote crystallization of PLA matrix upon heat-treatment if a significant interaction between glass fibers and PLA is achieved, i.e., if the surface of the glass fibers is modified and functionalized prior to the preparation of the composite.

**Figure 2:**
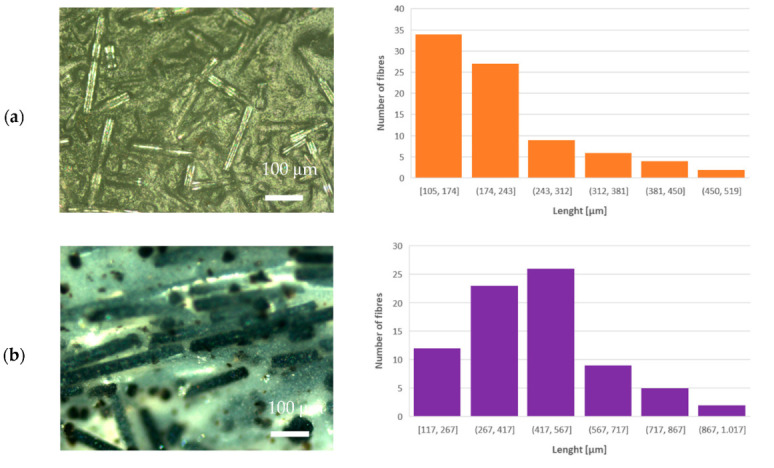

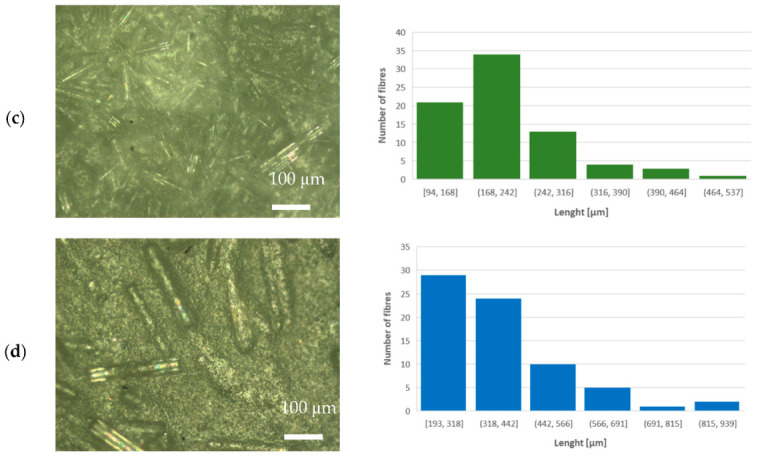
Optical microscope images (**left-hand side**) and length distribution of milled fibers (**right-hand side**) of (**a**) PLA–WG, (**b**) PLA–IPG, (**c**) PLA–HYBG, and (**d**) PLA–ARG composites.

The morphology of the prepared PLA–glass composites was studied with optical and SEM microscopy. From the optical microscope images shown in the left-hand panels in Figure 2, it can be observed that glass fibers in all composites are embedded in the PLA matrix with random distribution and orientation. Note also that the length of the glass fibers differs slightly: ~450 μm for IPG, ~380 μm for ARG, and ~220 μm for WG and HYBG fibers; see the histograms in the right-hand panels in Figure 2. The mean length and mean diameter of the glass fibers are reported in Table 1.

**Figure 3:**
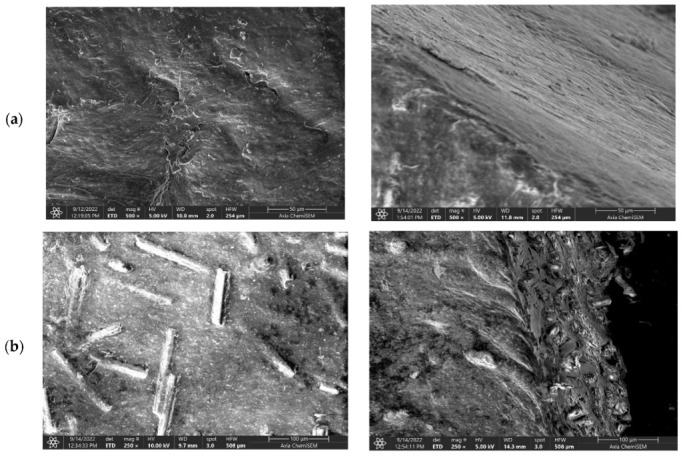

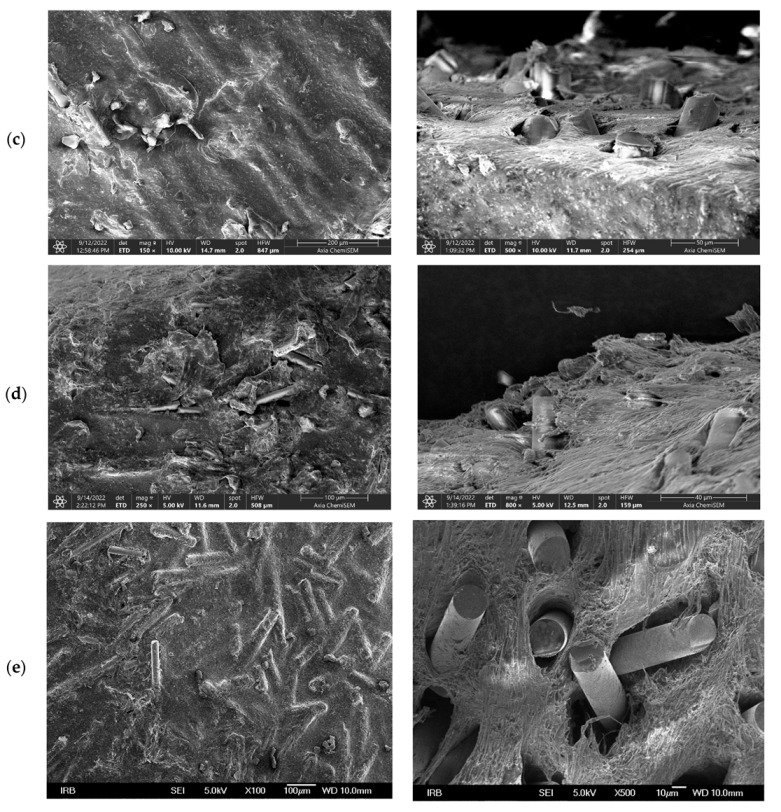
SEM images of surface (**left-hand side**) and cross-section (**right-hand side**) of (**a**) neat PLA, (**b**) PLA–WG, (**c**) PLA–IPG, (**d**) PLA–HYBG, and (**e**) PLA–ARG composites.

The SEM images of the surface and cross-section areas of the neat PLA and all PLA–glass fiber composites are shown in Figure 3. The neat PLA exhibits a smooth surface without specific morphological features. On the other hand, all four composites show very similar surface morphology as well as cross-section structure with glass fibers uniformly and randomly distributed through the bulk of the composites; see Figure 3b–e. Interestingly, it seems that the composition of the glass fibers, although being very different, i.e., borosilicate- vs. iron-phosphate-based, has very little if any influence on the formation and structure of the composite material. The same holds for the aspect ratios of the glass fibers, which vary from 15 for ARG and HYBG to 23 for WG and IPG; see Table 1. In particular, the images of the cross-section reveal that all types of glass fibers form a loose bond with the PLA matrix. Although the observed SEM images can result from the mechanical cross-sectioning itself, it should be noted that the surface of the glass fibers in this study was not modified to improve the bonding interaction with the polymer as in [7]. Therefore, somewhat poorer interaction between the glass fibers and PLA matrix could be expected in our composites. 

### 3.2. Thermal Properties of PLA–Glass Fiber Composites

**Figure 4:**
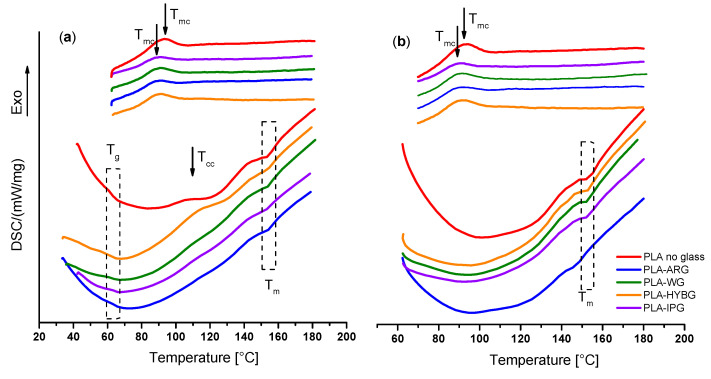
DSC curves for the neat PLA and PLA–glass composites in (**a**) first and (**b**) second heating-cooling cycle.

The DSC profiles of the neat PLA and PLA–glass fiber composites during heating and cooling are shown in Figure 4. In the DSC curve of the neat PLA, 3 thermal events are observed in the 1st heating run: glass transition (*T_g_*) at 62 °C, cold crystallization (*T_cc_*) at 110 °C, and melting (*T_m_*) at 155 °C; see Figure 4a. The observed temperatures correspond well to the literature data [15,19,20]. On the other hand, in the subsequent cooling run only a broad exothermic peak with a maximum at 95 °C related to melt crystallization (*T_mc_*) can be observed [19]. 

The DSC curves of the PLA–glass composites, irrespective of the type of glass fibers, show very similar features to those of the neat PLA in the first heating run, indicating that the addition of glass fibers does not affect glass transition or melting temperature. This result agrees well with the literature and is expected since the size of glass fibers is too macroscopic to affect the mobility of the polymer chain segment [15]. However, a shift in the crystallization temperature for all composites toward lower values upon cooling, accompanied by a decrease in the peak intensity, indicates that the crystallization of the melt occurs later. This signifies that all glass fibers have an inhibitory effect on the crystallization of PLA. The reason for this phenomenon could be due to two factors: (i) the lack of strong interaction between PLA matrix and glass fibers that could provide nucleation sites and/or (ii) a large amount of glass fibers (50 wt%) that prevents the growth of large crystalline domains. This corroborates the XRD results for the heat-treated PLA–glass composites, which show no crystallization in the PLA matrix upon heating, cf. Figure 1. Indeed, it is possible that in the heat-treated PLA–glass composite, a small amount of nanocrystalline sites is nucleated nonhomogenously and that was not sufficient to have a significant diffraction intensity. Interestingly, in the second heating run, the exothermic peak related to crystallization is absent in all samples, PLA and PLA–glass ceramics, suggesting that further crystallization is prevented while cooling in the 1st and 2nd run exhibits identical features.

### 3.3. Electrical Properties of PLA–Glass Fiber Composites

**Figure 5:**
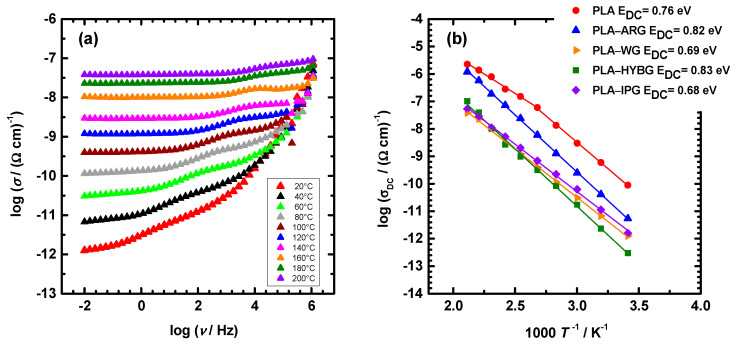
(**a**) Conductivity spectra at different temperatures for PLA–WG composite and (**b**) Arrhenius plot of DC conductivity for all composites.

The electrical properties of PLA–glass fiber composites were studied with impedance spectroscopy over a wide frequency and temperature range. Figure 5a displays conductivity spectra at different temperatures for PLA–WG sample, which is representative of all prepared composites. In these spectra, three characteristic features are visible: (i) a frequency-independent region at low frequencies corresponding to the DC conductivity, (ii) a step-like feature in the middle frequency range, and (iii) an increase in conductivity (dispersion) at lower temperatures and high frequencies. While the DC conductivity originates from the translational motions of charge carriers, the conductivity dispersion is related to their correlated short-range transport and, naturally, shifts to higher frequencies with temperature. On the other hand, the step-like feature of conductivity in the middle frequency range is related to the relaxation at the interfaces between regions of different conductivity [21], i.e., PLA matrix and glass fibers. This result indicates that the PLA matrix in the composites is not continuous but is strongly intersected with the glass fibers which block the charge carrier transport. It is worth noting that the conductivity spectra of the neat PLA do not show such spectral features in the middle frequency range (not shown here). In these conductivity spectra, only conductivity plateau (i) and dispersion (iii) are visible, which is in line with the homogenous continuous PLA matrix.

Generally, neat PLA is known to have excellent insulating properties such as low conductivity and high breakdown strength [22]. Indeed, the DC conductivity of PLA in our study is 9.0 × 10^−11^ (Ω cm)^−1^ at 20 °C which is consistent with the literature data [23]. It has also been reported that the insulating properties of the neat PLA deteriorate upon heating above 70 °C due to poor thermal resistance [22]. Similarly, our study reveals that the temperature dependence of the DC conductivity of the neat PLA exhibits two regions in the Arrhenius plot; see Figure 5b. The first region corresponds to the temperature range up to 100 °C, where linear temperature dependence is visible, while the second region is characterized by nonlinear changes in conductivity. This leads us to infer that the deterioration of the electrical properties of industrial-grade PLA used in our study occurs at higher temperatures, above 100 °C, which could be related to the presence of the calcite and talc that are used as fillers. With the addition of glass fibers, the DC conductivity of PLA–glass fiber composites significantly decreases; see Figure 5b. At 20 °C, the DC conductivity decreases up to two orders of magnitude, having values of 5.4 × 10^−12^ (Ω cm)^−1^ for PLA–ARG, 1.5 × 10^−12^ (Ω cm)^−1^ for PLA–IPG and PLA–WG, and 3.0 × 10^−13^ (Ω cm)^−1^ for PLA–HYBG. It is very likely that the observed variations in the DC conductivity of composites originate from the differences in the glass composition. However, it should be noted that these values are significantly lower than that of PLA. It is also particularly interesting to observe that the DC conductivity of all PLA–glass fiber composites exhibits Arrhenius behavior, i.e., linear temperature dependence over the entire temperature range. This result implies that the PLA–glass fiber composites are stable at much higher temperatures than neat PLA. Therefore, it can be concluded that the addition of all types of glass fibers not only improves the insulating properties by decreasing the conductivity but also extends the temperature range in which the composites are thermally stable.

## 4. Conclusions

In this work, we present the preparation of PLA–glass composites using different types of glass fibers (three commercial silicate/borosilicate-based and one laboratory-made iron-phosphate-based) and technical grade PLA in a weight ratio of 1:1. The structural, morphological, thermal, and electrical properties of all PLA–glass composites were found to be very similar and basically independent of the compositions and aspect ratios of the glass fibers, which varied from 15 to 23. All prepared PLA–glass composites contain homogeneously distributed and randomly oriented glass fibers. In comparison with the neat PLA, these materials show higher mechanical strength and thermal stability as well as better insulating properties. Therefore, these composites could be suitable for small-scale disposable applications in outdoor and indoor building applications where the in situ degradation of the material is targeted. 

## Figures and Tables

**Table 1:** The producer company, composition and mean length and diameter of glass fibers.

Glass Fibres	Company	Composition	Mean Length (μm)	Mean Diameter (μm)	Length/Diameter
ARG	Nippon Electric Glass Co.	Alkali resistant glass (SiO_2_-based glass containing ZrO_2_)	380	25	15
WG	Kelteks d.o.o.	E-glass (SiO_2_-B_2_O_3_-based glass)	216	10	22
HYBG	Hybon^TM^ 2002 Nippon Electric Glass Co.	E-glass (SiO_2_-B_2_O_3_-based glass)	222	15	15
IPG	laboratory-made	40Fe_2_O_3_-60P_2_O_5_	451	20	23

## Data Availability

Not applicable.
